# Clinical Significance of Combined Detection of CCL22 and IL‐1 as Potential New Bronchial Inflammatory Mediators in Children's Asthma

**DOI:** 10.1002/iid3.70043

**Published:** 2024-11-07

**Authors:** Lei Cui, Xiaozhen Song, Yanping Peng, Min Shi

**Affiliations:** ^1^ Department of Pediatrics People's Hospital of Xiangxi Tujia and Miao Autonomous Prefecture, First Affliated Hospital of Jishou University Jishou China

**Keywords:** asthma, CCL22, IL‐1

## Abstract

**Backgrounds:**

Severe asthma is a significant health burden because children with severe asthma are vulnerable to medication‐related side effects, life‐threatening deterioration, and impaired quality of life. However, there is a lack of data to elucidate the role of inflammatory variables in asthma. This study aimed to compare the levels of inflammatory factors in serum and sputum in children with acute and stable asthma to those in healthy children and the ability to predict clinical response to azithromycin therapy.

**Methods:**

This study recruited 95 individuals aged 1−3 years old and collected data from January 2018 to 2020. We examined serum and sputum inflammatory factors and constructed the least absolute shrinkage and selection operator (LASSO) model. Predictive models were constructed through multifactor logistic regression and presented in the form of column‐line plots. The performance of the column‐line diagrams was measured by subject work characteristics (ROC) curves, calibration plots, and decision curve analysis (DCA). Then, filter‐paper samples were collected from 45 children with acute asthma who were randomly assigned to receive either azithromycin (10 mg/kg, *n* = 22) or placebo (*n* = 23). Pretreatment levels of immune mediators were then analyzed and compared with clinical response to azithromycin therapy.

**Results:**

Of the 95 eligible participants, 21 (22.11%) were healthy controls, 29 (30.53%) had stable asthma, and 45 (47.37%) had acute asthma. The levels of interferon‐γ (IFN‐γ), tumor necrosis factor‐a (TNF‐α), chemokine CCL22 (CCL22), interleukin 12 (IL‐12), chemokine CCL4 (CCL4), chemokine CCL2 (CCL2), and chemokine CCL13 (CCL13）were significantly higher in the acute asthma group than in the stable asthma group. A logistic regression analysis was performed using CCL22 and IL‐1 as independent variables. Additionally, IFN‐γ, TNF‐α, IL‐1, IL‐13, and CCL22 were identified in the LASSO model. Finally, we found that CCL22 and IL‐1 were more responsive in predicting the response to azithromycin treatment.

**Conclusion:**

Our results show that CCL22 and IL‐1 are both representative markers during asthma symptom exacerbations and an immune mediator that can predict response to azithromycin therapy.

## Graphical abstract

Clinical significance of combined detection of CCL22 and IL‐1 as potential new bronchial inflammatory mediators in children asthma.

## 1 Introduction

Asthma is a prevalent disease characterized by chronic inflammation of the lower respiratory tract [1]. In the United States, approximately 24 million people are affected by asthma [2]. The prevalence of asthma has been increasing, with 3.1% of the US population affected in 1980 and 8.3% in 2016 [3]. Asthma is one of the most common chronic disorders in children, disproportionately affecting African Americans and those living in poverty [4]. Exacerbations of asthma result in lost school and work days, hospitalizations, emergency department visits, and over 3000 fatalities per year [5]. Therefore, identifying biomarkers of acute asthma is crucial for its diagnosis.

Individuals with upper airway inflammatory illnesses are more likely to have chronic lower airway inflammation [6]. Asthma imposes a significant financial burden, with the greatest economic impact being on individuals with poorly managed asthma and in low‐income nations [7, 8]. The airway epithelium is both a mediator and a target of inflammation in asthma, leading to remodeling and obstruction [9]. Airway wall remodeling may be triggered by immune cells producing proinflammatory cytokines, direct binding of IgE to smooth muscle cells or non‐immunological stimulation. Increasing research indicates that epigenetic mechanisms contribute to cell type‐specific illnesses and govern the connection between epithelium and subepithelial cells, initiating airway wall remodeling early in life [10]. Therefore, understanding the role of airway inflammation in asthma therapy is essential.

Asthma is a common respiratory disease that often develops in infancy due to airway sensitivity to common allergens, such as house dust mites, cockroaches, animal dander, fungi, and pollens. The selective proliferation of T cells, particularly of the T helper 2, is responsible for this condition. These T cells produce a cluster of cytokines encoded on chromosome 5q31‐33, including interleukins IL‐3, IL‐4, IL‐5, IL‐9, IL‐13, and granulocyte‐macrophage colony‐stimulating factor (GM‐CSF). Remodeling and inflammation are important components of asthma along with airway hyperresponsiveness.

Remodeling can occur in early childhood and does not necessarily follow inflammation, but rather parallels it. Airway reactivity is at least partially associated with bronchial inflammation. Increased airway smooth muscle in early childhood is associated with the late development of asthma and may be a link between inflammation and airway responsiveness [11, 12, 13]. In some children, hallmark mediators of inflammation (e.g., elevated levels of eosinophils) can distinguish asthma from other causes of asthma (e.g., viral infections) [14]. Jose et al. found that the relationship between inflammation and remodeling in children cannot be determined. Failure to demonstrate eosinophilic inflammation in the absence of remodeling is contrary to the hypothesis that inflammation causes these changes [15]. Uncontrolled maternal asthma during pregnancy exposes the developing fetus to inflammatory damage, which further increases the risk of childhood asthma unrelated to genetic susceptibility [16]. Saikumar Jayalatha et al. investigated that IL‐33 and its receptor IL‐1RL1 are considered putative biomarkers or targets for pharmacological intervention in asthma [17]. This process promotes the creation of chemoattractants, such as C−C motif chemokine 20 (CCL20), C−C motif chemokine 19 (CCL19), and C−C motif chemokine 27 (CCL27) [18, 19, 20, 21, 22]. The effect of treatment is largely independent of the presence of the most common pathogenic airway bacteria or viruses, suggesting that the drug's immune‐modulatory properties may play a role and that the individual child's immune response may determine and predict clinical response [23, 24, 25, 26]. Cytokines and chemokines mediate the immune response in the airways, but the current understanding of local immune mediator release during childhood acute asthma exacerbations is based primarily on stimulated in vitro airway models and airway lavage techniques that introduce unknown dilutions of airway secretions [27, 28, 29, 30, 31, 32].

Azithromycin has a relatively good therapeutic effect in the treatment of asthma. In a recent randomized controlled trial of oral azithromycin, it was demonstrated that in children aged 1−3 years with recurrent asthma‐like symptoms, the duration of attacks with asthma‐like symptoms was significantly reduced by 60% compared with placebo [33]. Interestingly, the therapeutic effect is largely independent of the presence of the most common pathogenic airway bacteria or viruses, suggesting that this effect may be mediated in part by the immunomodulatory properties of the drug and that the immune response in an individual child can determine and predict the clinical response. There is a need to screen regulatory factors to predict response to treatment to limit the use of antibiotics to children who are likely to benefit from them [34, 35, 36].

This study aims to compare inflammatory factor levels in serum and sputum in children with acute and stable asthma versus healthy children, analyze important inflammatory factors using a clinical prediction model, and assess the value of these mediators in predicting azithromycin treatment response. Considering that the immune response to the airways is mainly mediated by cytokines and chemokines, we also compared the levels of immune mediators in secretions and serum after azithromycin intervention and evaluated the value of these mediators in predicting response to azithromycin treatment.

## 2 Materials and Methods

### 2.1 Ethics Approval and Consent to Participate Section

This study was authorized and approved by the ethical committee and institutional review board (IRB) of the First Affiliated Hospital of Jishou University (JS20112231). Informed consent was obtained from all subjects and/or their legal guardian(s). The research adhered to the Helsinki Declaration and CONSORT standards.

### 2.2 Patient

This cross‐sectional, single‐center study recruited 95 individuals aged 1−3 years old from January 2018 to 2020. The children visited the study clinic nine times over 4 years and received urgent care during severe asthma episodes. Inclusion criteria are eligible children between the ages of 1 and 3 years with a physician's diagnosis of asthma. The diagnosis of stable asthma was the absence of new‐onset asthma in the last 3 months [37]. The subjects with acute asthma were recruited soon after presentation to our hospital with an acute exacerbation of asthma, as described previously [37, 38, 39]. Study doctors with pediatric expertise examined the children during acute care visits and followed standard operating procedures [33].

The sample size was determined to be 40 patients per group with *a* = 0.05 and *b* = 80%, with 5 participants lost to follow‐up. Each group ultimately had 40 participants. Each episode of asthma‐like symptoms between the ages of 1−3 years was randomly assigned to either a 3‐day course of azithromycin oral solution, 10 mg/kg per day, or a matching placebo. Episodes with clinical symptoms of pneumonia or blood CRP levels exceeding 476 nmol/L (50 mg/L) were excluded. The primary outcome was the diary‐verified duration of symptoms after commencing medication. This analysis is based on the data set from the original experiment [40].

### 2.3 Assay for Systemic and Airway Inflammatory Cytokines

At 2 years of age, upper airway epithelial lining fluid was collected from the nasal mucosa of the child during asymptomatic periods, as well as during episodes of asthma‐like symptoms at acute care visits from 0 to 3 years of age. This means that the samples could have been obtained both before and after the asymptomatic period sample [41]. Samples from the azithromycin versus placebo study were collected before randomization and drug administration. At 1 month of age, a filter‐paper collection approach was used to collect the upper airway epithelial lining fluid, which has been previously successful in analyzing immune mediator profiles in newborns [42, 43]. Filter‐paper strips (Accuwik Ultra, fibrous hydroxylated polyester sheets, cat no.SPR0730, Pall Life Sciences, Portsmouth, Hampshire, UK) were placed bilaterally in the anterior inferior turbinate of the nasal cavity and removed after 2 min of absorption, then instantly frozen at −80°C. To account for seasonal changes in upper airway immune mediator levels, sex, age, and sampling season were recorded [44]. The date of extraction from filter sheets, as well as the date and batch of analysis, were noted throughout laboratory analyses.

Throughout laboratory analyses, we recorded the date of extraction from filter sheets, as well as the date and batch of analysis. In both blood and sputum supernatant, we identified systemic or proinflammatory cytokines (C‐reactive protein [CRP], TNF‐α, IL‐1, and IL‐6), TH1/TH2‐like cytokines (IFN‐γ and IL‐5), and M1/M2‐macrophage‐like cytokines (TNF‐α, IL‐1, IL‐6, IL‐8, CCL17, and CCL‐22). Serum was retrieved from peripheral venous blood collected in Vacutainer tubes (BD Biosciences, San Jose, CA, USA) by centrifugation for 10 min at 3000 rpm, 4°C (H2050R, cence, Changsha, China). Enzyme‐linked immunosorbent assay (ELISA) reagent kits (R&D Systems Inc., Minneapolis, USA) were used to evaluate serum CRP, IL‐1, TNF‐α, IFN‐γ, IL‐5, IL‐8, IL‐6, CCL22, and CCL17 levels, as indicated by the manufacturer. The MILLIPLEX MAP Human Cytokine/Chemokine Magnetic Bead Panel Kit (EMD Millipore Corporation, Billerica, MA, USA) was used to assess IL‐1, TNF‐α, IFN‐γ, IL‐5, IL‐8, IL‐6, and CCL22 levels in sputum supernatant. If the cytokine concentration fell below the lower limit of detection of the test, the concentration was reduced to half the lower limit of detection [45].

### 2.4 Daily Prospective Monitoring of Acute Asthma Symptoms

Diary cards were utilized since infancy to document the burden of asthma‐like symptoms between visits, as previously established. Any symptom that significantly affects the child's breathing, such as wheezing or whistling noises, shortness of breath, or a persistent unpleasant cough that interferes with the child's sleep or activities, was classified as asthma‐like symptoms. Symptoms were recorded daily as separate components of wheezing, dyspnea, and cough, with any component constituting an asthma‐like day. An episode of asthma‐like symptoms in a child was defined as at least 3 consecutive days of symptoms. It is important to note that this study excluded samples from episodes with clinical signs of pneumonia or croup [46].

### 2.5 Least Absolute Shrinkage and Selection Operator (LASSO) Regression Prediction Model

The LASSO method was employed to minimize multivariate data and identify risk indicators for osteoporosis following asthma rupture. Non‐zero LASSO regression coefficients were utilized in the training set, and a prediction model was created by performing multiple logistic regression analyses on the given variables in the LASSO regression model. For each characteristic, the odds ratio (OR), 95% confidence interval (CI), and *p*‐value were determined, using two‐tailed statistical significance thresholds. Sociodemographic variables, as well as illness and treatment‐related factors, were included in the model if their *p*‐value was less than 0.05. A calibration curve was generated to assess the calibration of the nonadhesive coating nomogram, and the Harrell c index was derived to measure the nonadhesive coating nomogram's discriminative power. Decision curve analysis was conducted to identify clinically significant noncompliance nomograms and calculate the net benefit. The diagnostic efficacy of the clinical factor model was evaluated using the relevant ROC curve (AUC) from the training and validation toolbox [47, 48, 49, 50, 51].

### 2.6 Statistics

The study utilized the median and standard deviation to summarize the raw levels of immune mediators (pg/mL) during both the asymptomatic and symptomatic phases of asthma. Before further investigation, immune mediator concentrations were *z*‐scored to develop mediators with varying concentration ranges that were comparable in all aspects. Additionally, concentrations were total‐sum normalized per sample (immune mediator concentration/sum of all immune mediator concentrations in sample) to account for the high within‐sample collinearity of immune mediator levels, which was likely caused by varying amounts of nasal secretion collected on the filter papers. The association between immune mediator levels was measured and presented in hierarchical clustering heatmaps. The *χ* ^2^ test was employed to compare dichotomous variables. Linear mixed effect models were used to compare immune mediator levels during asthma‐like symptom episodes to asymptomatic periods, with mediator levels as response variables and clinical status (i.e., during or outside episodes) as the explanatory variable. The model included sample factors such as gender, age, and season, as well as the date of filter paper extraction, the date of analysis, and the batch of analysis. To account for repeated evaluations in samples acquired during bouts of asthma‐like symptoms, the model incorporated individual participant identification as a random variable. The data were presented as a ratio of mediator concentrations during asthma‐like symptoms versus asymptomatic periods, with 95% CIs and both nominal and false discovery rate (FDR) adjusted *p*‐values.

To avoid the possibility of multiple testing, we employed a principal component analysis (PCA) to decompose the signal from the 18 mediators into three independent principal components (PCs) that capture the overall variation in mediator levels. We then compared the PC scores during asthma‐like symptoms versus asymptomatic periods and included PCs as an explanatory variable in linear mixed‐effect models. The total treatment impact of azithromycin on episode length was evaluated using a Poisson regression model with a log link, consistent with the methods used in the original investigation. To investigate the influence of immunological mediator levels on azithromycin treatment response, we utilized linear mixed‐effect models with episode length as the outcome variable, treatment arm (azithromycin or placebo) as the explanatory variable, and mediator levels as an interaction term. Additionally, the online data addition adjusted for the outcomes of viral and/or bacterial airway infections. The results are presented as a factor change in treatment impact per rise in the standard deviation of immune mediator concentration in samples taken during acute asthma episodes.

To investigate the predictive capacity of immune mediator levels in a clinical context where data from only a single person would be available, unadjusted subanalyses were performed on raw immune mediator levels that were non‐*z*‐scored and non‐normalized. We evaluated the connection between a subset of single mediator levels and treatment response, as well as ratios between up‐ and downregulated immune mediators, as a simple alternative to normalization. All data analysis was conducted using the statistical software R v3.4.0 (R Core Team, 2015) and the add‐on package lme4.

To identify risk markers for osteoporosis during asthma rupture, the LASSO technique was utilized to minimize multivariate data. Non‐zero LASSO regression coefficients were employed in the training set, and multiple logistic regression analysis was performed on chosen variables in the LASSO regression model to produce a prediction model. The characteristics OR with 95% CI and *p*‐value were used, with statistical significance levels with two tails being utilized. Sociodemographic characteristics, as well as illness and treatment‐related variables, were included in the model with a *p*‐value less than 0.05. A calibration curve was created to examine the prediction nomogram's calibration, and the Harrell c index was derived to measure the prediction nomogram's discriminative power. Decision curve analysis was considered to uncover clinically useful prediction nomograms to determine net benefit. The diagnostic efficiency of the clinical factor model was assessed using the appropriate ROC curve from the training and validation toolbox (AUC). Another researcher used similar tactics to reproduce the findings. A statistically significant difference was defined as *p* < 0.05 [47, 48, 49, 50, 51].

## 3 Results

### 3.1 Characteristics of the Subject

Table 1 displays the characteristics of the 95 eligible children in our sample. Of these, 22.11% had health control, 30.53% had stable asthma, and 47.37% had acute asthma. In comparison to stable asthma, acute asthma exhibited a considerably lower ratio of forced expiratory volume in 1 s and forced vital capacity (FEV1/FVC%), inhaled corticosteroids (ICS), and atopy. However, there was no significant difference between stable and acute asthma in terms of severe yearly exacerbation frequency, maintenance of oral corticosteroid, oral corticosteroid dosage, long‐acting muscarinic antagonist, and theophylline.

**Table 1:** Characteristics of the healthy controls and subjects with stable and acute asthma.

	Health control	Stable asthma	Acute asthma	*p* value
*N*	21 (22.11)	29 (30.53)	45 (47.37)	
Sex, M (%)	11 (52.38)	20 (68.96)	25 (55.56)	0.323
Age, months	36.5	39.25	37.1	
BMI (kg/m^2^), mean + SD	28.61 ± 2.18	27.69 ± 3.69	28.15 ± 5.11	0.234
Asthma duration—years	N/A	26.54 ± 16.48	27.88 ± 12.98	0.112
Severe annual exacerbation frequency	N/A	1.02 ± 0.59	2.61 ± 0.11	0.322
Maintenance oral corticosteroids—%	N/A	25.99 ± 3.89	35.62 ± 6.59	0.112
Oral corticosteroid dose—mg	N/A	10.00 ± 4.25	12.62 ± 4.29	0.093
Long‐acting β2‐agonist—%	N/A	100	100	
Long‐acting muscarinic antagonist—%	N/A	38.56 ± 3.59	45.25 ± 2.86	0.324
Leukotriene receptor antagonist—%	N/A	48.62 ± 4.29	52.66 ± 4.15	0.212
Theophylline—%	N/A	14.36 ± 5.64	23.62 ± 4.85	0.342
Ex‐smoker—%	14.52 ± 2.61	23.62 ± 10.25	36.55 ± 10.54	0.423
Atopy, *n* (%)	11.02 ± 10.59	32.89 ± 11.25	45.61 ± 12.25	0.001
FEV1, % predicted	98.01 ± 8.95	84.28 ± 7.62	62.88 ± 8.11	0.822
FEV1/FVC%	85.75 ± 5.66	72.48 ± 6.25	61.28 ± 5.48	0.011
ICS, mg/day	N/A	5360.16 ± 120.25	1548 ± 158.64	0.023

### 3.2 Inflammatory Cytokine Levels in the Blood and Sputum

The inflammatory cytokine levels were significantly up‐ and downregulated during acute and stable asthma episodes. Table 2 indicates that serum IL‐5, IL‐8, TNF‐α, CCL22, IL‐12P70, CCL4, CCL2, and CCL13 levels were considerably greater in acute asthma than in stable asthma. However, other inflammatory cytokines showed no difference between acute and stable asthma. Additionally, we examined the levels of inflammatory cytokines in sputum and found that IL‐5, IL‐6, IL‐8, TNF‐α, IFN‐γ, CCL17, CCL22, CXCL10, CCL4, CCL2, IL‐4, IL‐13, and CCL26 were significantly lower in stable asthma than in acute asthma (Table 3).

**Table 2:** Serum airway inflammatory cytokine in asthma patients.

	Health control	Stable asthma	Acute asthma	*p* value
CRP, pg/mL	0.25 ± 0.18	1.25 ± 0.26	1.95 ± 0.36	0.229
IL‐1β, pg/mL	0.42 ± 0.03	0.51 ± 0.02	0.85 ± 0.11	0.584
IL‐5, pg/mL	1.59 ± 0.25	6.25 ± 0.89	21.68 ± 4.25	0.011
IL‐6, pg/mL	0.25 ± 0.03	1.58 ± 0.59	2.99 ± 0.15	0.056
IL‐8, pg/mL	10.25 ± 1.25	18.25 ± 2.68	29.65 ± 2.48	0.025
TNF‐α, pg/mL	2.59 ± 0.65	10.25 ± 2.61	19.25 ± 4.26	0.001
IFN‐γ, pg/mL	1.15 ± 0.85	3.26 ± 0.95	8.25 ± 2.06	0.256
CCL17, pg/mL	8.61 ± 0.95	10.21 ± 2.61	19.64 ± 2.85	0.065
CCL22, pg/mL	398.25 ± 12.62	486.25 ± 35.61	493.54 ± 29.64	0.002
IL‐12p70, pg/mL	0.59 ± 0.05	1.25 ± 0.36	1.95 ± 0.28	0.036
CXCL10, pg/mL	2853.61 ± 236.15	3256.24 ± 365.15	4865.27 ± 623.15	0.224
CCL4, pg/mL	224.61 ± 11.25	356.14 ± 16.56	425.61 ± 34.28	0.012
CCL2, pg/mL	52.81 ± 11.20	136.39 ± 22.61	325.15 ± 36.58	0.032
CCL13, pg/mL	10.39 ± 2.61	15.68 ± 3.64	19.84 ± 4.20	0.014
IL‐4, pg/mL	0.25 ± 0.05	0.78 ± 0.04	0.95 ± 0.08	0.059
IL‐13, pg/mL	10.29 ± 2.68	16.28 ± 0.24	20.64 ± 5.41	0.078
CCL11, pg/mL	100.19 ± 12.65	186.29 ± 25.68	223.61 ± 43.28	0.085
CCL26, pg/mL	5.26 ± 1.02	7.58 ± 2.64	10.22 ± 1.84	0.099

**Table 3:** Sputum airway inflammatory cytokine in asthma patients.

	Health control	Stable asthma	Acute asthma	*p* value
CRP, pg/mL	0.38 ± 0.02	2.61 ± 0.58	3.68 ± 0.81	0.258
IL‐1β, pg/mL	0.65 ± 0.11	0.95 ± 0.25	1.25 ± 1.15	0.114
IL‐5, pg/mL	2.22 ± 0.55	7.15 ± 1.15	18.64 ± 2.68	0.025
IL‐6, pg/mL	0.58 ± 0.22	1.98 ± 0.21	3.65 ± 1.25	0.018
IL‐8, pg/mL	12.68 ± 1.25	18.25 ± 2.68	29.65 ± 2.48	0.032
TNF‐α, pg/mL	1.28 ± 0.62	13.88 ± 2.61	22.84 ± 1.21	0.048
IFN‐γ, pg/mL	2.28 ± 0.64	4.85 ± 0.58	9.18 ± 1.22	0.025
CCL17, pg/mL	5.69 ± 2.64	13.75 ± 2.61	23.68 ± 2.64	0.022
CCL22, pg/mL	442.61 ± 52.12	556.27 ± 11.25	658.98 ± 56.98	0.011
IL‐12p70, pg/mL	0.25 ± 0.15	0.62 ± 0.02	1.25 ± 0.86	0.268
CXCL10, pg/mL	2953.65 ± 125.84	3958.75 ± 265.18	4582.62 ± 362.85	0.01
CCL4, pg/mL	202.25 ± 12.62	396.51 ± 45.62	495.28 ± 58.27	0.015
CCL2, pg/mL	48.62 ± 18.64	89.64 ± 5.64	92.51 ± 7.89	0.035
CCL13, pg/mL	12.68 ± 2.18	19.58 ± 4.27	26.35 ± 10.28	0.031
IL‐4, pg/mL	0.38 ± 0.04	0.89 ± 0.15	1.18 ± 0.24	0.025
IL‐13, pg/mL	10.29 ± 2.68	16.28 ± 0.24	20.64 ± 5.41	0.034
CCL11, pg/mL	86.21 ± 3.58	175.62 ± 18.67	220.85 ± 15.94	0.058
CCL26, pg/mL	3.75 ± 0.85	8.15 ± 0.98	10.22 ± 1.84	0.024

What's more, as shown in Table 4, the levels of IL‐1β (*p* = 0.025), IL‐5 (ratio of acute asthma episodes to stable asthma episodes (*p* = 0.024), IL‐6 (*p* = 0.018), TNF‐α (*p* = 0.011), IFN‐γ (*p* = 0.011), CCL17 (*p* = 0.025), CCL22 (*p* = 0.021), IL‐12p70 (*p* = 0.015), C−X−C‐motif chemokine 10 (CXCL10) (*p* = 0.048), CCL4 (*p* = 0.023), CCL2 (*p* = 0.048), CCL13 (*p* = 0.025), IL‐4 (*p* = 0.018), IL‐13 (*p* = 0.024), and CCL11 (*p* = 0.028) were found to be downregulated in the serum of patients with stable asthma compared to those with acute asthma.

**Table 4:** Upper airway immune mediator levels during episodes of acute asthma compared to stable periods.

	Serum				Sputum			
Immune mediator	Ratio of immune mediator levels (acute asthma/stable asthma)	CI95	*p* value	Corrected *p* value	Ratio of immune mediator levels (acute asthma/stable asthma)	CI95	*p* value	Corrected *p* value
CRP	1.56	0.25−0.69	0.625	0.251	1.41	0.68−2.61	0.621	0.025
IL‐1β	1.67	0.89−1.25	0.025	0.015	1.32	0.12−4.55	0.251	0.011
IL‐5	3.45	0.95−1.66	0.024	0.024	2.61	0.25−1.25	0.221	0.005
IL‐6	1.89	0.98−1.96	0.018	0.015	1.86	0.15−1.66	0.362	0.014
IL‐8	1.62	0.75−1.18	0.054	0.032	1.62	0.35−1.85	0.058	0.032
TNF‐α	3.95	0.62−1.86	0.011	0.005	1.64	0.62−2.11	0.025	0.024
IFN‐γ	2.83	0.58−1.12	0.015	0.025	1.89	0.42−1.11	0.011	0.014
CCL17	1.18	0.69−1.15	0.025	0.014	1.72	0.25−1.15	0.062	0.025
CCL22	1.01	0.65−1.26	0.021	0.022	1.18	0.25−1.25	0.022	0.029
IL‐12p70	1.56	0.68−1.65	0.015	0.011	2.02	0.26−1.62	0.021	0.014
CXCL10	1.49	0.75−1.58	0.048	0.025	1.16	0.36−1.22	0.028	0.058
CCL4	1.19	0.35−1.15	0.023	0.014	1.25	0.26−1.58	0.095	0.154
CCL2	2.39	0.24−1.89	0.048	0.025	1.03	0.24−1.36	0.028	0.025
CCL13	1.26	0.29−1.25	0.025	0.061	1.34	0.25−1.58	0.024	0.017
IL‐4	1.21	0.38−2.15	0.018	0.028	1.32	0.35−2.68	0.026	0.032
IL‐13	1.26	0.64−2.68	0.024	0.062	1.26	0.15−1.58	0.021	0.033
CCL11	1.2	0.98−3.62	0.028	0.015	1.25	0.62−2.68	0.012	0.385
CCL26	1.34	0.86−4.25	0.062	0.02	1.25	0.32−2.51	0.005	0.321

*Note:* False discovery rate adjusted *p*‐value.

Abbreviation: CI95, 95% confidence interval.

The serum levels of TNF‐α (*p* = 0.025), IFN‐γ (*p* = 0.011), CCL22 (*p* = 0.022), IL‐12p70 (*p* = 0.021), CXCL10 (*p* = 0.028), CCL2 (*p* = 0.028), CCL13 (*p* = 0.024), IL‐4 (*p* = 0.026), IL‐13 (*p* = 0.024), and CCL11 (*p* = 0.028) were found to be downregulated in stable asthma when compared to acute asthma.

### 3.3 Nomogram Development and Internal Validation

**Figure 1:**
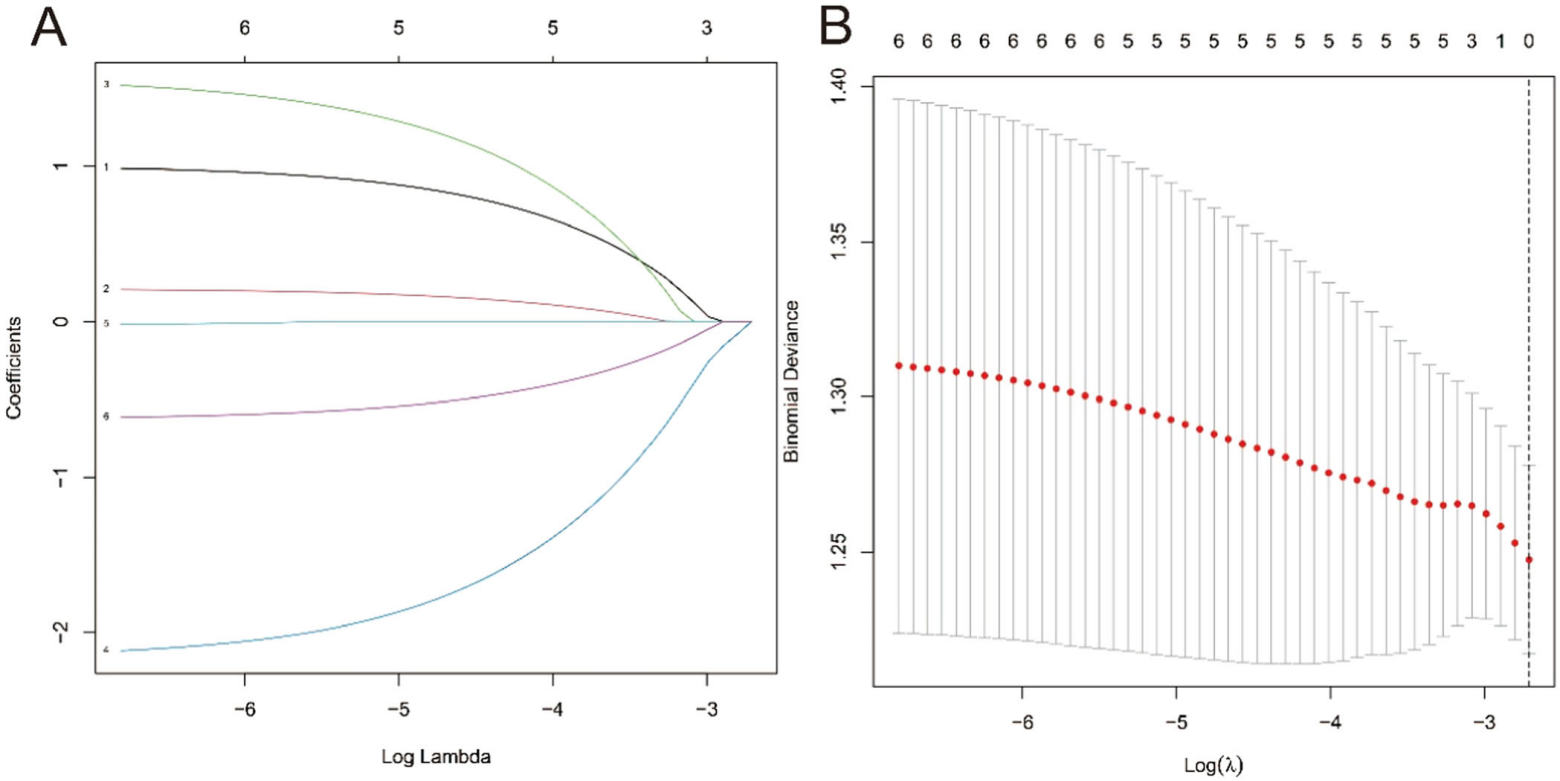
LASSO model in our studies (A, B).

A logistic regression analysis was conducted to examine the predictive ability of five components, namely IFN‐γ, TNF‐α, IL‐1, IL‐13, and CCL22. Nomograms were created based on the aforementioned independent predictors. The risk calibration curve for the non‐concordant isotype map demonstrated high agreement among patients (Figure 1). The training group showed a nomogram C‐index of 0.958 (95% CI: −1.22 to 1.25), indicating considerable discrimination. The breach risk graph indicated a high level of predictability.

**Figure 2:**
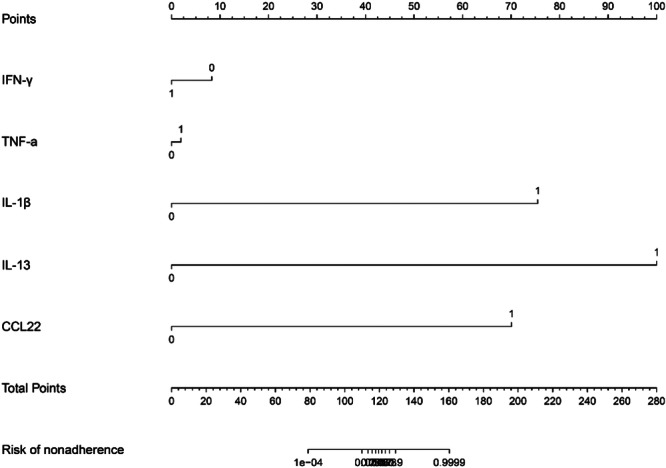
Line graph analysis of airway inflammation related asthma in infants.

**Figure 3:**
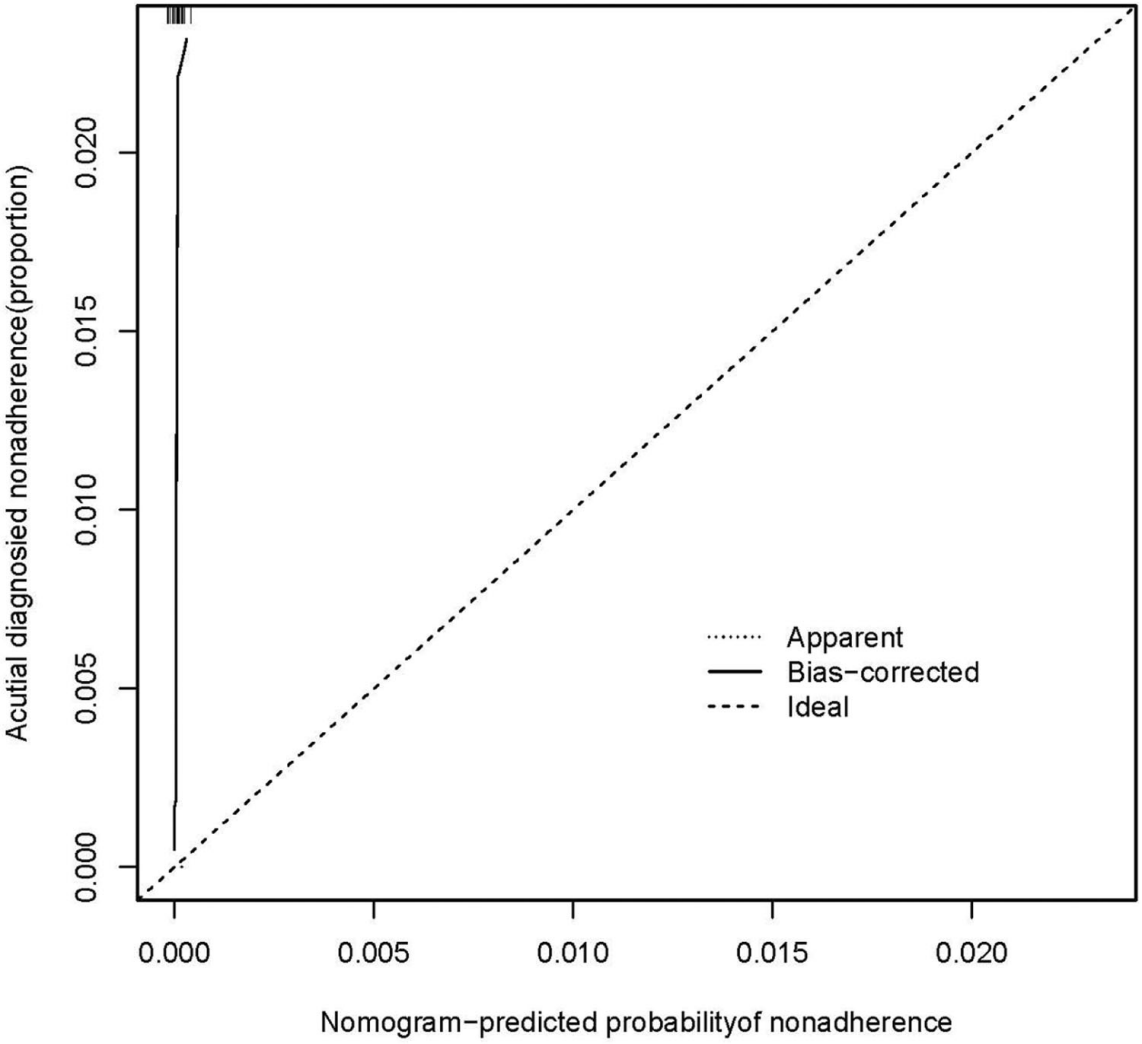
ROC curves of inflammatory cytokine with acute asthma and stable asthma signature.

Figure 3 displays the decision curve analysis for inflammatory cytokine with acute and stable asthma. The prediction nomogram was found to be superior to the strategy of using determinants of inflammatory cytokine with acute asthma and stable asthma, as indicated by the decision curves. The net benefit was equivalent over a range where both the patient and physician probability criteria were between 14% and 88%, with several overlaps (Figure 2). The ROC curves of inflammatory cytokines with acute asthma and stable asthma signatures are also shown in Figure 3. In the training set, the AUC of the nomogram in inflammatory cytokine with acute asthma and stable asthma was greater than that of the clinical factor model (*p* = 0.018, AUC = 0.995). Furthermore, there was a significant difference in the AUC nomogram in the validation set between the inflammatory cytokine with acute asthma and stable asthma and the clinical factors model (*p* = 0.036, AUC = 0.958). These results suggest that IFN‐γ, TNF‐α, IL‐1, IL‐13, and CCL22 are more accurate predictors of asthma diagnosis.

### 3.4 Upper Airway Immune Mediator Profiles Were Analyzed to Predict the Response to Azithromycin Treatment in Bouts of Acute and Stable Asthma

Filter‐paper samples were collected from 45 children with acute asthma who were randomly assigned to receive either azithromycin (*n* = 22) or placebo (*n* = 23). The number of missed episodes was similar in both treatment groups (*n* = 22 in the azithromycin group and *n* = 23 in the placebo group). The results showed that the average duration of symptoms after treatment was 3.15 days in the azithromycin group and 9.28 days in the placebo group, indicating a significant decrease of 61.02% (*p* < 0.01) in episode length due to azithromycin medication in this subgroup of data.

Table 5 presents the association between three immune mediators and the effect of azithromycin on symptom duration. CCL22 and IL‐1 were found to be significantly up‐ or downregulated during acute and stable asthma attacks.

**Table 5:** Treatment effect of azithromycin for episodes of acute asthma depending on upper airway immune mediator levels.

Immune mediator	Treatment effect (factor/increase SD of concentration)	CI95	*p* value	Concentration of no treatment effect (SD)
INF‐γ	1.25	0.22−2.62	0.85	< 0.62
TNF‐α	0.99	0.15−3.69	0.23	< −0.69
CCL22	3.25	1.00−2.69	0.02	> 0.15
IL‐1ß	1.56	0.25−1.02	0.02	< 1.25
IL‐13	0.25	0.26−1.25	0.29	> 0.85

*Note: p*‐value for interaction.

Abbreviations: CI95, 95% confidence interval; SD, standard deviation.

## 4 Discussion

This study represents the initial clinical inquiry into the roles of CCL22 and IL‐1 in acute asthma. Given the staggering number of individuals worldwide affected by asthma, exceeding 300 million, and the projected increase to 400 million by 2025, there is an urgent need for effective and sustainable treatments. Allergic asthma, which is impacted by immunological factors, accounts for 90% of pediatric asthma cases and 50% of adult asthma cases [52, 53, 54]. Our findings indicate that CCL22 and IL‐1 levels were significantly elevated in both acute and stable asthma groups compared to the control group.

Moreover, levels of CCL22 and IL‐1 were significantly higher in acute asthma compared to stable asthma [55]. The production of chemokines, particularly CCL22, plays a crucial role in regulating the migration of CC chemokine receptor 4 (CCR4)‐expressing immune cells to the airways in type 2 inflammatory airway disorders, such as allergic asthma. CCR4 is expressed by type‐2 inflammatory cells, including Th2 cells and innate lymphoid cells, which generate IL‐4, IL‐5, and IL‐13, and contribute to the etiology of allergic asthma [56, 57, 58]. In animal models of allergic airway disease, the genetic deletion of either CCR4 ligand (CCL17 or CCL22) reduced lung inflammation, Th2 infiltration, and airway hyperreactivity [59, 60]. Monoclonal antibodies targeting CCR4, such as mogamulizumab, have been approved in Japan for the treatment of adult T‐cell leukemia‐lymphoma [61]. CCL17 and CCL22 have been studied as potential therapeutic targets for type 2 inflammatory diseases, including asthma. However, it remains unclear whether STAT6 and/or NF‐κB influence the development of pulmonary CCL17 and CCL22 in the context of rhinovirus (RV) infection and asthma. Additionally, the results demonstrate that STAT6 regulates CCL17 and CCL22 in a context‐dependent manner [62]. Mirko et al. revealed that during cigarette smoke‐induced airway inflammation, CCR4‐expressing airway epithelial cells are the principal source of CCR4 ligands [63].

Viral‐induced exacerbations of asthma are associated with the production of IL‐1, which generates both Th1 and Th2 inflammation and causes neutrophil inflammation. IL‐1 may also enhance Th2‐type cytokine expression [64]. The activation of neutrophil chemotactic factors, IL‐33, and Muc5ac expression during viral stimulus‐induced aggravation requires IL‐1 signaling, indicating that IL‐1 plays a role in both neutrophilic and Th2 inflammation [65]. In humans, IL‐1 expression is associated with neutrophilic airway inflammation, disease severity, and steroid resistance. In mice, anti‐IL‐1 therapy reduced IL‐1 responses and the primary steroid‐resistant elements of illness, while IL‐1 treatment restored these characteristics [66]. Wei et al. identified that the IL‐1 signaling pathway signal transducers IL‐1 could be utilized as biomarkers for the pathophysiology of juvenile asthma and as potential treatment targets [67]. Therefore, dysregulation of IL‐1 and IL‐1 signaling contributes to illness pathogenesis, highlighting the paradox of IL‐1 therapeutic studies in asthma [68]. Our findings indicate that CCL22 and IL‐1 levels were higher in acute asthma than in stable asthma.

The chemokine CCL22 is produced primarily by dendritic cells (DCs) and regulates T reg migration. CCL22 controls T cell immunity by recruiting T regs to tumor tissues and by promoting the formation of DC‐T reg contacts in lymph nodes [69]. Baer et al. found that somatic CCL22 mutations illustrate a unique mechanism of tumor formation in which gain‐of‐function chemokine mutations promote tumorigenesis [70]. Ren et al. found that in the presence of CCL22, CCR3‐positive fibroblast‐like synoviocytes (FLS) upregulate CCL22 and S100A12 driving a potential feedforward proinflammatory mechanism distinct from canonical CCL22 and CCR3 pathways [71]. Previous studies have found that CCL22 also plays an important role in asthma. Chiu et al. found that low mother‐to‐child Th2‐associated chemokine CCL22 levels appear to be inversely related to mite sensitization and the risk of asthma development in early childhood [72]. Christian et al. investigated that asthma‐like symptoms in young children are orchestrated by the local airway immune response and CCL22 may predict the response to azithromycin treatment [40]. In our study, we also found that CCL22 and IL‐1 may serve as independent predictors of acute asthma.

Sputum inflammatory factors also play a very important role in lung diseases. Thangakunam et al. found that induced sputum may reflect changes in cytokine milieu in sarcoidosis [73]. Osika et al. also found that impairment of IL‐6 expression in sputum may represent an important component of the excessive inflammatory response observed in cystic fibrosis [74]. Elevated levels of nociceptin and inflammatory and remodeling markers were found in non‐eosinophilic asthma [75]. Tanaka et al. investigated that spontaneous sputum can provide helpful information on airway inflammatory phenotyping in patients with asthma [76]. In addition, in children with asthma, sputum inflammatory phenotypes are variable in both stable and exacerbation phases, in contrast to data in adults [77]. In our study, we also found that sputum levels of inflammatory factors like IL‐5, IL‐6, and so forth also appeared to be significantly increased in asthmatics.

Azithromycin is an antibiotic. Since its discovery, it has been approved by the FDA for use in respiratory infections (e.g., pneumonia), genitourinary infections (e.g., chlamydia), and intestinal infections (e.g., typhoid fever), and it has also been widely used for malaria [29]. Jagat et al. found that the use of azithromycin in children with poorly controlled asthma resulted in improved asthma control and reduced exacerbations [30]. Peter et al. also found that adults with persistent symptomatic asthma experience fewer asthma exacerbations and improved quality of life when treated with oral azithromycin for 48 weeks [31]. Taylor et al. found that in patients with persistent uncontrolled asthma, azithromycin reduced airway *H. influenzae* load compared with placebo but did not change the total bacterial load. Macrolide resistance increased, reflecting previous studies [32]. However, a systematic review investigated the efficacy and safety of azithromycin in asthma and found no beneficial clinical outcome of azithromycin in asthma control, and we propose that further prospective cohorts are warranted [33].

Macrolide antibiotics have demonstrated efficacy in both eosinophilic and non‐eosinophilic subtypes, with antibacterial, antiviral, and anti‐inflammatory activities. Systematic reviews of randomized controlled trials have shown that macrolides are beneficial for asthma symptoms. However, conclusions about their effects on other endpoints, such as exacerbations, cannot be drawn due to a lack of data, heterogeneity of findings, and poor research design and sample size [78]. Azithromycin has been found to decrease airway pressure in adults with uncontrolled chronic asthma, and to reduce influenzae burden when compared to a placebo, although overall bacterial load is not affected. Macrolide resistance has increased, highlighting the need for more research into the effectiveness of nonantibiotic macrolides as a long‐term treatment for those with chronic uncontrolled asthma [79]. Treatment with oral azithromycin for 48 weeks has been shown to reduce asthma exacerbations and improve quality of life in adults with chronic symptomatic asthma, as well as in children with poorly controlled asthma [80]. In Chinese patients, azithromycin may enhance PEF, ACT, and FEV1%, but not patient quality of life, and may be useful as an asthma adjuvant treatment [81]. However, a comprehensive examination has indicated that azithromycin has no positive clinical benefits in asthma control [82]. Our research has observed that azithromycin plays a crucial role in reducing the advancement of asthma.

## 5 Limitation

Some limitations in our study. First, the number of cases included in our study was limited, and the duration of follow‐up was short; further supplementation of the number of cases and duration of follow‐up is needed in future studies to increase the depth and reliability of the study. Second, in this study, we mainly studied several indicators of inflammatory factors and also used the LASSO regression model to analyze the changes in the levels of inflammatory factors, but there may be other serological indicators, that may need to be added in future studies. In addition, our sample size is estimated to be 40 patients per group, but for the control group, the number of cases did not reach 40, which may be related to the short enrollment time and the loss of follow‐up of patients in the control group. So in future studies we will increase the sample size of the control group.

## 6 Conclusion

In conclusion, our findings indicate that CCL22 and IL‐1 levels are markedly higher in acute asthma compared to stable asthma and also an immune mediator that can predict response to azithromycin therapy.

## Author Contributions

**Lei Cui** and **Xiaozhen Song:** conceptualization. **Yanping Peng** and **Min Shi:** data curation. **Yanping Peng** and **Min Shi:** formal analysis. **Yanping Peng** and **Min Shi:** funding acquisition. **Yanping Peng** and **Min Shi:** investigation. **Yanping Peng** and **Min Shi:** methodology. **Lei Cui** and **Xiaozhen Song:** project administration. **Lei Cui** and **Xiaozhen Song:** resources. **Lei Cui** and **Xiaozhen Song:** software. **Lei Cui** and **Xiaozhen Song:** supervision. **Lei Cui** and **Xiaozhen Song:** validation. **Lei Cui** and **Xiaozhen Song:** visualization. **Lei Cui** and **Xiaozhen Song:** writing–original draft. **Lei Cui, Xiaozhen Song, Yanping Peng,** and **Min Shi:** writing–review and editing. We all agree to publication.

## Ethics Statement

This study was authorized and approved by the ethical committee and institutional review board (IRB) of the First Affiliated Hospital of Jishou University (JS20112231). The research adhered to the Helsinki Declaration and CONSORT standards.

## Consent

Informed consent was obtained from all subjects and/or their legal guardian(s).

## Conflicts of Interest

The authors declare no conflicts of interest.

## Supporting information

Supporting information.

## Data Availability

The data used to support the findings of this study are included in the article.
